# Self-Reported Food Hypersensitivity: Prevalence, Characteristics, and Comorbidities in the Norwegian Women and Cancer Study

**DOI:** 10.1371/journal.pone.0168653

**Published:** 2016-12-16

**Authors:** Monika Dybdahl Jakobsen, Tonje Braaten, Aud Obstfelder, Birgit Abelsen

**Affiliations:** 1 Department of Community Medicine, UiT The Arctic University of Norway, Tromsø, Norway; 2 Center for Care Research, The Norwegian University of Science and Technology (NTNU), Gjøvik, Norway; 3 Department of Health and Care Sciences, UiT The Arctic University of Norway, Tromsø, Norway; 4 Norwegian Centre of Rural Medicine, Department of Community Medicine, UiT The Arctic University of Norway, Tromsø, Norway; Indiana University Bloomington, UNITED STATES

## Abstract

**Background:**

This study aims to investigate the prevalence of self-reported food hypersensitivity, (SFH), the characteristics of women with SFH, and whether SFH is associated with multiple health complaints among the participants of the Norwegian Women and Cancer study (NOWAC).

**Methods:**

We conducted a cross-sectional study among 64,316 women aged 41–76 years. The women were randomly selected from the Norwegian Central Person Register. Information on SFH and all covariates except age and place of residence was collected by questionnaires in 2002–2005.

**Results:**

The prevalence of SFH in our study sample was 6.8% (95% confidence interval: 6.7–7.0). Logistic regression analysis showed a negative association between SFH and age (odds ratio [OR] 0.97). The odds of SFH increased among women living in or near urban centers, women with more than 9 years of education, women who did not have full-time work, women who had experienced poor economic conditions in childhood, those living without a partner, and those who did not consume alcohol or smoke (OR varied from 1.10 to 1.70). Women with a low body mass index had higher odds of SFH (OR 1.37) than those with a moderate body mass index. SFH was positively associated with poor self-perceived health (OR 2.56). The odds of SFH increased with the number of concurrent health complaints, with an OR for 5–6 comorbidities of 4.93.

**Conclusion:**

We found an association between SFH, poor health, and different socio demographic and lifestyle characteristics. Women with SFH had increased odds of reporting multiple health complaints.

## Introduction

Food hypersensitivity is a collective term for all adverse reactions to food [1]. In the medical literature, food hypersensitivity is categorized into allergic and non-allergic food hypersensitivity; the latter group has also been referred to as food intolerance [1]. Persons who self-report food hypersensitivity may have various diagnoses of allergic- or non-allergic food hypersensitivity from conventional practitioners, or they may have self-diagnosed or alternative medicine-diagnosed food hypersensitivity.

The field of food hypersensitivity is one in which much debate is taking place, and it seems to be characterized by a lack of solid scientific knowledge. It is a common perception that the prevalence of self-reported food hypersensitivity (SFH) is increasing [2, 3], and even though some studies on subgroups of SFH support this perception, this apparent increase is not well documented [4, 5]. Furthermore, studies show a disparity between the prevalence of food hypersensitivity based on self-report and the prevalence based on medical tests [6, 7]. This may imply that food hypersensitivity is overreported, but may also be related to the food hypersensitivity tests, which can have weaknesses or be laborious [2]. Moreover, some perceive SFH as an excuse for dieting [8], while others feel that some persons are misled by alternative medicine to believe they are hypersensitive to some foods [3]. Still others accept individuals’ perception of their symptoms as being food-induced and emphasize the need for further research on the biological causes of food hypersensitivity [9]. Some of the suggested biological causes include the introduction of new foods, excessive hygiene, changes in the consumption of fatty acids, and changes in the microbiota of the gut [2, 10].

In order to give adequate health care attention to this heterogeneous group it is important that we learn more about the prevalence and the characteristics of persons with SFH. Food hypersensitivity in adults is often a long-term condition, and such conditions require continuous self-care work. [11–13]. The capacity to manage this type of ongoing self-care work, such as implementing a restricted diet, will be influenced by the person’s resources and by eventual comorbidities [11–13]. It is therefore useful to know more not only about the prevalence of SFH, but also the characteristics of persons with SFH, and whether SFH is associated with other lasting health complaints.

A systematic review of prevalence studies concluded that the prevalence of SFH varied both between studies and between countries, with prevalence estimates ranging from 3% to 35% for any food [6]. One of the studies included in this review presented prevalence estimates of 4.6% in Spain, 19.1% in Australia, and approximately 16% in Norway [14]. The more recent Euro Prevall study underpins this heterogeneity, with self-reported adverse reactions to food in women varying from 5–8% in Lithuania, Greece, Poland, and Spain, to 30% in Germany [15].

Studies addressing the characteristics of persons with SFH indicate a female predominance [16, 17]. They further suggest that young women with higher education more often report adverse reactions to food than older women with lower education [17]. Another study indicated that individuals with SFH are more often absent from work, but that only 2% of that study sample felt that their income had been affected due to food-attributed symptoms [18]. A study from a clinical setting reported that fewer persons with SFH than controls consumed alcohol, but persons with SFH had the same degree of smoking and physical activity as controls [19]. A study on food allergy, one of the subgroups of SFH, suggested that it is more often reported among city residents [20]. Studies on other subgroups of SFH, such as individuals with Crohn’s disease and gluten sensitive persons, indicated that they have a lower body mass index (BMI) than controls [21–23]. Other studies on celiac disease and irritable bowel syndrome showed that these diseases led to an increased burden on the subject’s partner [24, 25], which may contribute to a lower degree of couple relationships among people with SFH. Previous studies documented the association between socioeconomic conditions in childhood and different health outcomes in adulthood [26], and this association may apply to SFH as well.

Ambiguous results have been reported concerning the overall health status of persons with food hypersensitivity [27]. In Poland, people with SFH reported a poorer overall health status than controls, while the opposite was observed in Spain, and in the UK and the Netherlands no differences were found [27].

According to studies from clinical settings, persons with unexplained or perceived food hypersensitivity report multiple health complaints more often than controls, including fatigue, musculoskeletal pain (among others back pain), depression, and fibromyalgia [9, 28]. Increased risk of depression and fatigue may also be related to untreated celiac disease [29], and celiac disease is associated with immune mediated diseases including autoimmune thyroid diseases [30].

In the present study, our first aim was to investigate the prevalence of SFH using a large representative sample. Our second aim was to illuminate the characteristics associated with SFH. Based on former studies, we hypothesized that SFH would be associated with young age, living in urban areas, having high education level, having low employment status, poor economy in childhood, not living with a partner, low alcohol consumption, and low BMI. We did not expect to find an association between SFH and income, smoking, or physical activity. Our third aim was to test the hypotheses that SFH is associated with poor health and with reporting multiple health complaints.

The large representative sample available to us was the Norwegian Woman and Cancer study (NOWAC). This sample included women 41–76 years, and as a result of this, the study was delimited to women belonging to this age span.

## Materials and Methods

### Data source

The NOWAC study is a population-based prospective cohort study, which was initially established to explore oral contraceptive use and other risk factors for breast cancer. The study has also been used to explore other cancer- and diet-related hypotheses, and has been described in detail elsewhere [31]. The NOWAC sample is randomly selected from the Norwegian Central Person Register, which contains information about all residents in Norway. Between 1991 and 2007 approximately 172,000 women aged 30–70 years were included in the study (overall response rate 52.7%). All women have given written informed consent to participate, and the Regional Ethical Committee for Medical Research Ethics and the Norwegian Data Inspectorate have approved the NOWAC study.

Participants recruited in the 1990s received a follow-up questionnaire in 2002–2005, and data from these questionnaires were used in the present cross-sectional analysis. Altogether 81,065 follow-up questionnaires were mailed, of which 64,316 were returned. All analyses in the present study were based on group anonymous data.

The follow-up questionnaire included basic questions on the use of oral contraceptives, reproductive history, family history of breast cancer, smoking, alcohol consumption, anthropometry, physical activity, and socioeconomic factors [32], as well as questions about health, health complaints, diet, and SFH. The question about food hypersensitivity was initiated by the following formulation: “Do any of the following conditions influence your diet?” Among the possible responses was the alternative “have allergy/intolerance”. We categorized women who ticked “have allergy/intolerance” as having SFH, and all others as not having SFH.

### Statistical analysis

Data were analyzed using STATA version 14. Age was included as a continuous variable, since there was a linear association between age and SFH. Other study variables were categorized as follows: SFH (yes/no), place of residence (central–not central (reference)), duration of education (≤9 (reference),10–12,13–16, ≥17 years), employment status (full-time work (reference), not full-time work), economic conditions in childhood (good (reference), poor), partner status (living with a partner (reference), not living with a partner), alcohol consumption (<0.1, 0.1–4.9 (reference), 5.0–9.9, ≥10 g/day), smoking status (never (reference), former, current), BMI (<20, 20–24.9 (reference), ≥25 kg/m^2^) and self-perceived health (good (reference), poor).

The place of residence variable is based on Statistics Norway’s classification of centrality. “Central” includes municipalities with a regional center and a population of at least 50000, as well as municipalities that are within 75 minutes (90 for Oslo) travel from this regional center. The smoking variable was constructed based on the following two questions: “Have you during your life smoked more than 100 cigarettes?” (yes/no), and “Do you smoke daily now?” (yes/no). The question concerning self-perceived health is initiated with “Do you perceive your health as:”, and the respondents can tick off for”very good”, “good”, “poor” or “very poor”. Six possible health complaints, which had comprehensive interaction, were merged into one variable with the following categories: no comorbidities, muscle pain (myalgia) only, fibromyalgia/fibrositis only, low back pain only, depression only, hypothyroidism only, chronic fatigue only, two concurrent comorbidities, three concurrent comorbidities, four concurrent comorbidities, and five–six concurrent comorbidities.

Prevalence is presented as percentages, with 95% confidence intervals (CI). Characteristics of women with and without SFH are presented as means or percentages, along with associated p-values based on the Mann-Whitney test or the Chi-square test. Logistic regression analysis was conducted to investigate the association between SFH and participant characteristics, and odds ratios (OR) and p values are presented. The dependent variable was SFH, and the independent variables were age, place of residence, duration of education, employment status, economic conditions in childhood, partner status, alcohol consumption, smoking status, BMI, and self-perceived health. Due to an observed interaction effect, a term for interaction between smoking and alcohol consumption was included in the model. Self-reported physical activity level (“today” on a scale from 1 to 10) and household income were initially included in the model, but were not associated with SFH, and thus were excluded from the analysis.

A second logistic regression analysis was performed to investigate the association between SFH and reporting other health complaints. The same variables mentioned above were included, but self-perceived health was replaced with the health complaints variable.

Some of the variables had missing values (see S1 Appendix for the distribution of missing values). The depression and hypothyroidism variables had a relatively high percentage of missing values, and were recoded the following way: respondents with negative or missing answers who answered the subsequent question about when the depression or hypothyroidism started were coded as having depression or hypothyroidism, while the rest were coded as not having depression or hypothyroidism. After this recoding, multiple imputation was conducted, using the chained equations procedure in Stata, and 20 datasets were created. The multiple imputation procedure included all variables involved in the logistic regression analyses, plus variables perceived as predictive of missing values (number of children, physical activity level, and income). After the imputation procedure, means of observed and completed data were compared, showing small differences. Results from the logistic regression analyses based on complete-case data showed results that were similar to those from the logistic regression analyses based on imputed data (see tables B and C in S2 Appendix for logistic regression based on complete case data).

## Results

The study sample included 64,316 women aged 41–76 years (mean age 57.1 years), and 6.8% (95% CI: 6.7–7.0) had SFH. The mean age for women with SFH was lower than for women without SFH (Table 1). Women living in or near urban centers had higher odds of SFH than women living in less central parts of the country (Table 2). Women with more than 9 years of education and those without full-time work had increased odds of SFH. Respondents who had experienced poor economic conditions in childhood had higher odds of SFH, and this association was independent of age. A larger percentage of women with SFH were not living with a partner, had never smoked, did not consume alcohol, or was former smokers and non-consumers of alcohol. Moreover, women with a low BMI (<20) had a higher risk of SFH than women with a moderate BMI (20–24.9 kg/m^2^), and the SFH group contained more women with poor self-perceived health (Table 2).

**Table 1:** Characteristics of women with and without self-reported hypersensitivity (SFH), the Norwegian Women and Cancer study (complete case data).

	With SFH (n = 4,405)	Without SFH (n = 59,911)	p*
Age (years, mean)	56.1	57.1	<0.001
Place of residence (%)			
Central	59.0	55.8	
Not central	41.0	44.2	<0.001
Duration of education (years, %)			
≤9	22.9	28.4	
10–12	34.1	34.2	
13–16	28.4	25.9	
≥17	14.6	11.5	<0.001
Employment status (%)			
Full-time work	37.9	42.3	
Not full-time work	62.1	57.7	<0.001
Economic conditions in childhood (%)			
Good	70.0	73.2	
Poor	30.1	26.8	<0.001
Partner status (%)			
Living with partner	75.1	78.8	
Not living with partner	24.9	21.2	<0.001
Alcohol consumption (g/day, %)			
<0.1	22.1	19.9	
0.1–4.9	54.3	55.2	
5.0–9.9	15.5	16.5	
≥10	8.1	8.4	<0.002
Smoking status (%)			
Never	41.1	39.1	
Former	36.3	36.5	
Current	22.6	24.4	<0.008
Body mass index (kg/m^2^, %)			
<20	7.1	4.9	
20–24.9	46.3	47.8	
≥25	46.6	47.3	0.018
Self-perceived health (%)			
Good	82.0	92.3	
Poor	18.0	7.7	<0.001

* P-value: Mann-Whitney or Chi-square test.

**Table 2:** Odds ratios (OR) with p values of self-reported food hypersensitivity by participant characteristics, the Norwegian Women and Cancer study (imputed data).

	OR	p
Age (years)	0.97	<0.001
Place of residence		
Not central (ref.)	1.00	
Central	1.10	0.003
Duration of education (years)		
≤9 (ref.)	1.00	
10–12	1.28	<0.001
13–16	1.45	<0.001
≥17	1.69	<0.001
Employment status		
Full-time work (ref.)	1.00	
Not full-time work	1.30	<0.001
Economic conditions in childhood		
Good (ref.)	1.00	
Poor	1.20	<0.001
Partner status		
Living with partner (ref.)	1.00	
Not living with partner	1.26	<0.001
Smoking status among non-alcohol consumers		
Never (ref.)	1.00	
Former	1.35	<0.001
Current	0.87	0.152
Smoking status among alcohol consumers (≥0.1 g/day)		
Never (ref.)	1.00	
Former	0.86	<0.001
Current	0.79	<0.001
Body mass index (kg/m^2^)		
<20	1.37	<0.001
20–24.9 (ref.)	1.00	
≥25	0.98	0.467
Self-perceived health		
Good (ref.)	1.00	
Poor	2.56	<0.001

The analysis which included the health complaints variable showed increased odds of SFH among women with muscle pain (myalgia), fibromyalgia/fibrositis, back pain, depression, hypothyroidism, or chronic fatigue syndrome, and the odds of SFH increased gradually with increasing number of concurrent comorbidities (Table 3). A testing of the association between SFH and the number of health complaints indicated an OR of 1.42 for each additional health complaint (p <0.001).

**Table 3:** Odds ratios (OR) with p values of self-reported food hypersensitivity by comorbidity* in the Norwegian Women and Cancer study (imputed data).

Comorbidities	OR**	p
No comorbidities (ref.)	1.00	
Muscle pain (myalgia) only	1.80	<0.001
Fibromyalgia/fibrositis only	1.72	0.001
Back pain only	1.24	0.002
Depression only	1.30	<0.001
Hypothyroidism only	1.61	<0.001
Chronic fatigue only	2.55	<0.001
2 concurrent comorbidities	1.16	<0.001
3 concurrent comorbidities	3.02	<0.001
4 concurrent comorbidities	4.12	<0.001
5–6 concurrent comorbidities	4.81	<0.001

* The six comorbidities considered were muscle pain (myalgia), fibromyalgia/fibrositis, back pain, depression, hypothyroidism and chronic fatigue.

**Adjusted for age, place of residence, duration of education, employment status, economic conditions in childhood, partner status, alcohol consumption, smoking status, and body mass index.

## Discussion

### Main findings

We found a prevalence of SFH of 6.8% among adult women in the NOWAC study. The odds of SFH decreased with age, and was increased among women who lived in or near urban centers, those who had more than 9 years of education, those without a full-time job, with poor economic conditions in childhood, those living without a partner, non-drinkers, never smokers, former smokers who did not consume alcohol, and women with low BMI. However, we did not observe a significant association between SFH and income or physical activity level. SFH was associated with poor self-perceived health, and with reporting multiple health complaints.

The prevalence of SFH in the present study was relatively moderate compared to other studies. One reason for this may be related to how the question on food hypersensitivity was formulated. Respondents who reported having food allergy/intolerance were defined as having SFH, and all others as not having SFH. Consequently, some participants who did not answer the question may have been misclassified as not having SFH.

The age of the women in our study sample is relatively high compared to other studies [14, 15], which may have contributed to the moderate prevalence we observed, since older persons tend to have lower odds of reporting food hypersensitivity [17]. Another explanation may be that the NOWAC study contains a large random sample of women and does not specifically focus on food hypersensitivity, thus minimizing the risk of food hypersensitive persons being overrepresented.

A larger proportion of young women, women in or near urban centers, and women with a high education level had SFH, which is congruent with studies from France and Germany [17, 20]. One may speculate whether this is due to a greater awareness of, or focus on, food hypersensitivity in these groups.

The fact that women who did not consume alcohol had higher odds of SFH is also consistent with other studies [19]. One possible explanation is that some persons are hypersensitive to alcoholic beverages [33], and there may be a correlation between being hypersensitive to food and to alcoholic beverages. It is also documented that alcohol may enhance hypersensitive reactions to food [34].

The association we observed between not smoking and SFH is not in line with a small study on SFH [19], but it is in line with another study that reported low tobacco use among patients with celiac disease [21]. The fact that former smokers who were non-drinkers had increased odds of SFH may indicate a change to a healthier lifestyle. This may be related to a general increased focus on healthy lifestyle, or personal experiences of alcohol and smoking as being detrimental to health.

The present study showed a negative association between SFH and full-time work, which persisted after controlling for self-perceived health. This is in line with studies which indicated more absence from work among individuals with SFH [18], as well as studies concluding that persons with chronic illness have less labor participation than others, even after controlling for physical disabilities [35].

Women who had poor economic conditions in childhood had increased odds of SFH. This finding may be seen in relation to the relatively well documented association between socioeconomic conditions in childhood and different health outcomes in adulthood [26]. It has been suggested that early socioeconomic environment may influence diet, cognitive and emotional development, or changes in gene expression that can influence adult health [26].

The present study also indicated an association between SFH and living without a partner, which may be related to the increased partner burden that has been identified in subgroups of SFH [24, 25]. More generally, studies have concluded that persons with health challenges are less likely to be married, and suggest that this can be related to strains on the relationship [36].

The association between SFH and low BMI is in line with other studies on persons who avoid gluten or have Crohn’s disease [21–23]. Previous studies have also suggested an increased risk of inadequate nutrition in subgroups of SFH [37, 38], and the nutritional state among persons with SFH seems to be worth further investigation.

The present study indicated an association between SFH, poor self-perceived health, and one or more concurrent comorbidities. These findings are consistent with the majority of other studies [9, 28, 29, 39, 40], and indicate that a significant subgroup of women with SFH have poor health and comorbidities.

### Strengths and limitations

The major strength of this study is the large and representative study sample, which was randomly selected among all women residing in Norway. An examination of external validity revealed no notable sources of selection bias or differences between the source population and NOWAC study participants, except for a somewhat higher education level [32]. There is a limited amount of representative studies of this magnitude, and as far as we know, the present study is the first to examine SFH using a representative sample of this size.

One weakness of this study is that the NOWAC questionnaires were not originally designed to deal with our research question. Another weakness is that the sample did not include men or younger women, thus our results cannot be generalized to the general adult population. A sample including younger women would have shown if the linear association we observed between age and SFH also applies to women in general. A sample including both sexes would have made it possible to compare the prevalence of SFH by sex, and may have revealed whether the findings related to women also applied to men.

The data used in the present study is from questionnaires sent in 2002–2005, which may be considered a weakness, since changes in prevalence may have occurred since then. As previously mentioned, the possible increase in SFH prevalence is not well documented [4, 5] and requires further research. Another weakness is that the analysis did not include all health complaints that may be related to SFH, for example asthma, allergic rhinitis, and eczema, which are conditions that often accompany food allergies [41].

Other weaknesses of the present study are related to the missing values. Missing values on the depression and hypothyroidism variables were recoded to ‘no’, based on the assumption of a connection between not responding and not having these conditions. Although this may have led to misclassification, we believe that recoding is preferable to other approaches. For other missing values, multiple imputation was conducted in order to preserve information from subjects with missing values [42]. Multiple imputation relies on the assumption that values are missing at random [42], but one can never conclude this with certainty. For example, some respondents may omit an answer because they find the categories inappropriate, and these people may tend to belong to particular groups.

## Conclusions

The present study indicates a relatively low prevalence of SFH in Norwegian women, and should be taken into account when debating the extent of SFH. The study also showed an association between SFH, poor health and reporting several health complaints. This indicates that a subgroup of women with SFH may need relatively complex health care interventions. In addition, poor health and having to manage additional health complaints may influence one’s capacity to implement a restricted and sometimes challenging diet, and a poorly implemented diet may affect health. Food hypersensitivity, be it SFH or more specific food hypersensitivity, is a topic on which more research is required.

## Supporting Information

S1 Appendix Missing values. (DOCX)

S2 Appendix Logistic regression based on complete case data. (DOCX)
