# Supplementary material for: Self-Reported Food Hypersensitivity: Prevalence, Characteristics, and Comorbidities in the Norwegian Women and Cancer Study
Source: PLoS One. 2016 Dec 16;11(12):e0168653. doi: 10.1371/journal.pone.0168653 (PMC5161385; doi:10.1371/journal.pone.0168653)
Supplement: S1 Appendix — (DOCX) [file pone.0168653.s001.docx]

## **Missing values**

**Supplementary table A.** Distribution of missing values for the variables included in the

logistic regression analyses.

| **Variable** | **Missing values** |
| --- | --- |
| Self-reported food hypersensitivity | 0^1^ |
| Age | 0 |
| Place of residence | 0 |
| Education | 3,425 |
| Employment status | 618 |
| Economic conditions in childhood | 3,997 |
| Partner status | 312 |
| Smoking status | 1,841 |
| Alcohol consumption (g/day) | 3,123 |
| BMI | 2,639 |
| Self-perceived health | 2905 |
| Muscle pain (myalgia) | 0^1^ |
| Fibromyalgia/fibrositis | 0^1^ |
| Back pain | 0^1^ |
| Depression | 12,391 |
| Hypothyroidism | 15,957 |
| Chronic fatigue | 0^1^ |

^1^Women who ticked off for having the condition were interpreted as reporting the health complaint, and the rest as not reporting the health complaint.
