# Supplementary material for: Self-Reported Food Hypersensitivity: Prevalence, Characteristics, and Comorbidities in the Norwegian Women and Cancer Study
Source: PLoS One. 2016 Dec 16;11(12):e0168653. doi: 10.1371/journal.pone.0168653 (PMC5161385; doi:10.1371/journal.pone.0168653)
Supplement: S2 Appendix — (DOCX) [file pone.0168653.s002.docx]

## **Logistic regression based on complete case data**

**Supplementary table B.** Odds ratios (OR) with p values of self-reported food hypersensitivity by participant characteristics, the Norwegian Women and Cancer study (compete case data).

|  | | | **OR** | **p** |
| --- | --- | --- | --- | --- |
| Age (years) | | | 0.97 | <0.001 |
| Place of residence | | |  |  |
|  | Not central (ref.) | | 1.00 |  |
|  | Central | | 1.11 | 0.004 |
| Duration of education (years) | | |  |  |
|  | ≤9 (ref.) | | 1.00 |  |
|  | 10-12 | | 1.30 | <0.001 |
|  | 13-16 | | 1.43 | <0.001 |
|  | ≥17 | | 1.69 | <0.001 |
| Employment status | | |  |  |
|  | Full-time work (ref.) | | 1.00 |  |
|  | Not full-time work | | 1.28 | <0.001 |
| Economic conditions in childhood | | |  |  |
|  | | Good (ref.) | 1.00 |  |
|  | | Poor | 1.18 | <0.001 |
| Partner status | | |  |  |
|  | Living with partner (ref.) | | 1.00 |  |
|  | Not living with partner | | 1.27 | <0.001 |
| Smoking status among non-alcohol consumers | | |  |  |
|  | Never (ref.) | | 1.00 |  |
|  | Former | | 1.39 | <0.001 |
|  | Current | | 0.87 | 0.224 |
| Smoking status among alcohol consumers (≥0.1 g/day) | | |  |  |
|  | Never (ref.) | | 1.00 |  |
|  | Former | | 0.92 | 0.029 |
|  | Current | | 0.79 | <0.001 |
| Body mass index (kg/m^2^) | | |  |  |
|  | <20 | | 1.42 | <0.001 |
|  | 20-24.9 (ref.) | | 1.00 |  |
|  | ≥25 | | 0.99 | 0.859 |
| Self-perceived health | | |  |  |
|  | Good (ref.) | | 1.00 |  |
|  | Poor | | 2.57 | <0.001 |

**Supplementary table C.** Odds ratios (OR) with p values of self-reported food hypersensitivity by comorbidity in the Norwegian Women and Cancer study (complete case data).

| **Comorbidities** | **OR^1^** | **p** |
| --- | --- | --- |
| No comorbidities (ref.) | 1.00 |  |
| Muscle pain (myalgia) only | 1.77 | <0.001 |
| Fibromyalgia/fibrositis only | 1.80 | 0.001 |
| Back pain only | 1.22 | 0.009 |
| Depression only | 1.27 | 0.001 |
| Hypothyroidism only | 1.57 | <0.001 |
| Chronic fatigue only | 2.32 | <0.001 |
| 2 concurrent comorbidities | 2.05 | <0.001 |
| 3 concurrent comorbidities | 2.93 | <0.001 |
| 4 concurrent comorbidities | 4.18 | <0.001 |
| 5-6 concurrent comorbidities | 4.75 | <0.001 |

^1^Adjusted for age, place of residence, duration of education, employment status, economic conditions in childhood, partner status, alcohol consumption, smoking status, and body mass index.
